# Genetic Effects for Individual Honeybee Grooming Behavior in Response to Varroa Mites and Its Relationship with the Mite Infestation Levels of Honeybee Colonies

**DOI:** 10.3390/genes16070792

**Published:** 2025-06-30

**Authors:** Miguel Enrique Arechavaleta-Velasco, Laura Yavarik Alvarado-Avila, Claudia García-Figueroa, Francisco Javier Ramírez-Ramírez, Vicente Eliezer Vega-Murillo, Moisés Montaño-Bermúdez

**Affiliations:** 1 Centro Nacional de Investigación Disciplinaria en Fisiología y Mejoramiento Animal, Instituto Nacional de Investigaciones Forestales, Agrícolas y Pecuarias, Querétaro 76280, Mexico; alvarado.laura@inifap.gob.mx (L.Y.A.-A.); garcia.claudia@inifap.gob.mx (C.G.-F.); ramirez.javier@inifap.gob.mx (F.J.R.-R.); montano.moises@inifap.gob.mx (M.M.-B.); 2 Facultad de Medicina Veterinaria y Zootecnia, Universidad Veracruzana, Veracruz 91710, Mexico; vvega@uv.mx

**Keywords:** honeybee, *Varroa destructor*, grooming behavior, genetic effects, Varroa infestation levels

## Abstract

**Background/Objectives**: The objectives of this study were to identify the genetic effects involved in the expression of individual honeybee grooming behavior in response to *Varroa destructor* and to determine if there is an association between the expression of this behavior and the infestation levels of Varroa in the honeybee colonies. **Methods**: The study was conducted in a population of 112 colonies composed of six segregating genetic groups that were derived from two honeybee lines that were selected for high and low individual honeybee grooming behavior. The individual honeybee grooming behavior of 3974 workers from the 112 colonies was measured by the time it takes a bee to respond in performing grooming behavior after a mite was placed on her body. The population growth of Varroa in the colonies was measured over a period of six months. **Results**: Differences between the genetic groups were found in the expression of individual honeybee grooming behavior (*p* < 0.01). The distribution of means of the genetic groups fits a genetic additive and dominance effects model for the expression of individual honeybee grooming behavior (*r*^2^ = 0.95; *p* < 0.01). Differences between the genetic groups were found in the colony population growth of Varroa over a period of six months (*p* < 0.01). A positive correlation was found between the mean individual honeybee grooming behavior of the colonies and the Varroa level of infestation in the colonies (*r* = 0.57; *p* < 0.01). **Conclusions**: The results indicate that additive and dominance genetic effects are associated with honeybee individual grooming behavior and that this trait has an effect on the levels of Varroa infestation in the colonies.

## 1. Introduction

The mite *V. destructor* is the most serious problem that beekeeping faces worldwide. Varroa represents a serious threat to the survival of honeybee colonies and to honey production. The mite is a factor involved in colony losses in different regions of the world [1,2,3,4,5], and the honey production of infested colonies is significantly affected [6].

Honeybee colonies are treated with chemical products that give a certain degree of mite control. However, in the long term, the use of miticides can cause serious problems, since mite populations are able to develop resistance to chemical products [7,8,9,10,11,12,13], and the use of these products can leave chemical residues in honey and wax that affect both honeybees and humans [14,15,16,17,18,19,20,21,22].

Beekeeping needs ways of maintaining productive colonies with low levels of Varroa mite infestation. One option is the development of Varroa-resistant honeybee lines through breeding. The selection of genotypes that are able to maintain low levels of mite infestation will contribute to an integrated pest management approach that would allow beekeepers to keep healthier and productive colonies and would reduce the risks associated with the constant use of chemical products to control the mite.

Two breeding strategies can be followed to develop mite-resistant honeybee lines. One of these strategies consists of identifying colonies that have survived a Varroa mite infestation without treatment for several years and using them to develop resistant honeybee lines through breeding. The other strategy consists of developing resistant honeybee lines by selecting colonies that express the behavioral mechanisms that confer resistance to the bees against the mite.

Honeybees have mechanisms of resistance against the mite, which include grooming behavior, Varroa-specific hygienic behavior, the relative attractiveness of worker brood and adult bees to the mite, shorter capped brood periods, and host factors that affect mite fertility and reproduction [23,24,25,26,27,28,29,30].

The results of some studies indicate that grooming behavior is involved in the resistance of honeybee colonies against the mite [29,31,32,33,34].

Honeybee grooming behavior involves the removal of the mite from the body of an adult bee and is divided into autogrooming and allogrooming. In the first, a worker bee grooms herself with her legs to get rid of a mite, and, sometimes, the bee uses her mandibles to bite the mite; in the latter, the infested bee attracts other workers that remove the mite from her body with their mandibles [28,33,35,36].

Grooming behavior has been studied at the colony level [29,31,33,37,38] and at the individual honeybee level [31,39,40,41,42]. At the colony level, the expression of grooming behavior has been associated with low mite infestations and with a high proportion of damaged mites collected of the bottom boards of honeybee colonies [29,31,32,33,34]. However, no relation has been reported between the expression of individual honeybee grooming behavior and the infestation levels of Varroa in the colonies.

Individual grooming behavior in response to Varroa is studied under laboratory controlled conditions and is measured by recording the time it takes for a bee to respond in performing autogrooming behavior after a mite is placed on her body [39], by rating the intensity of the autogrooming response of worker bees that are artificially infested with a mite [31,40], and by recording if artificially infested bees dislodge the mites from their bodies by autogrooming [31,40].

Individual honeybee grooming behavior in response to Varroa is partially genetic in origin [31,39] and environmental effects that influence the expression of the trait have been identified [43,44].

A QTL for individual honeybee grooming behavior in response to Varroa mites, named *groom-1*, has been identified and mapped on honeybee chromosome five [39]. The 95% confidence interval for the position of the QTL contains a small number of candidate genes, which include *Atlastin*, *Ataxin*, and *Neurexin-1 (AmNrx-1)* [39]. The effect of the QTL *groom-1* and the candidate genes on the expression of grooming behavior was confirmed in studies conducted in other honeybee populations [41,42,45]. The genes *cytochrome P450 (CYP9Q3)*, and the *splicing factor 45* (*Spf45*), that are also located in the genomic region of the QTL, have been associated with the expression of the trait [45]. Other genes, which include the *dopamine receptor* (*Dop2*) and the *encoding odorant-binding proteins Obp4*, that are located in chromosome nine, and the genes *Obp14* and *Obp16*, located in chromosome fifteen, have also been associated with the expression of individual honeybee grooming behavior [45].

Even though a QTL and genes have been found to be associated with the expression of individual honeybee grooming behavior in response to Varroa, little is known about the genetic mechanisms underlying the expression of this behavior.

It is important to establish if individual honeybee grooming behavior is associated with the infestation levels of Varroa in the colonies and to understand the genetic mechanisms involved in the expression of this behavioral trait, in order to determine if individual honeybee grooming behavior in response to Varroa could be incorporated into breeding programs to develop mite-resistant honeybee genotypes.

The objectives of this study were to identify the genetic effects involved in the expression of individual honeybee grooming behavior in response to Varroa and to determine if there is an association between the expression of this behavior and the Varroa mite infestation levels of honeybee colonies.

## 2. Materials and Methods

### 2.1. Experimental Population

A high-grooming-behavior colony and a low-grooming-behavior colony were used as parental sources to establish an experimental population of honeybee colonies. These two colonies were obtained from a high-grooming-behavior honeybee line and a low-grooming-behavior honeybee line.

A parental queen was reared from the high-grooming-behavior colony and a parental queen was reared from the low-grooming-behavior colony. Each of the queens was artificially inseminated with the semen of three drones that were full sibs of each queen. This inbreeding step was performed to obtain more genetically uniform daughter queens reared from the parental queens.

**Figure 1:**
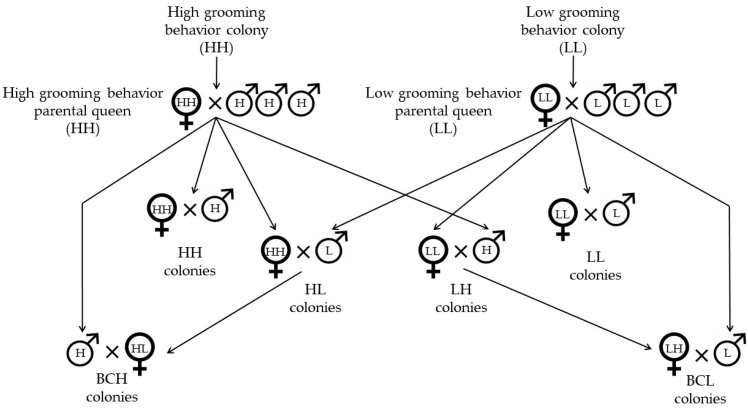
Mating scheme used to generate the experimental population that consisted of high-grooming-behavior colonies (HH), low-grooming-behavior colonies (LL), reciprocal F1 colonies (HL) and (LH), high-grooming-behavior backcross colonies (BCH), and low-grooming-behavior backcross colonies (BCL).

Queens and drones were reared from the two parental queens and, using instrumental insemination, a series of crosses were made to generate single-drone-inseminated queens that lead the colonies of an experimental population of 112 colonies that consisted of the following: high-grooming-behavior colonies HH (*n* = 18); low-grooming-behavior colonies LL (*n* = 19); reciprocal F1 colonies HL *(n* = 20) and LH (*n* = 18); high-grooming-behavior backcross colonies BCH (*n* = 18); and low-grooming-behavior backcross colonies BCL (*n* = 19) (Figure 1).

Each queen was introduced into a small colony made with two frames with sealed brood, two frames with honey, and approximately 2.0 kg of bees obtained from colonies treated against Varroa mites. The colonies were kept in five-frame single deep Jumbo-type hives in the same apiary and were managed in the same way during the time of the study.

### 2.2. Genetic Effects for Individual Honeybee Grooming Behavior

Individual honeybee grooming behavior was measured in the colonies by the time it takes a bee to respond in performing grooming behavior after a mite was placed on her body. The individual grooming behavior was evaluated 150 days after the experimental colonies were established to allow time for workers in the colony to be replaced by daughters of the single-drone-inseminated queens.

The behavioral assay was conducted in an observation chamber built with a plastic frame for the production of sections of comb honey. In one of the sections, an observation chamber (15.0 × 11.0 × 1.5 cm) was built. The chamber was covered by a transparent acrylic panel (15.0 × 11.0 × 0.1 cm) with a gate (4.0 × 4.0 cm) that was cut in the acrylic panel at the center of the lower border of the chamber to introduce bees into the observation area and to place the mites on the bees. Honeybee wax foundation was used to cover the bottom of the chamber. An acrylic strip (15.0 × 0.1 × 1.5 cm) was placed inside the observation chamber; and the strip was glued to a plastic pole (15.0 × 0.5 cm) that passed through a hole located at the center of the top border of the chamber, in order to move the acrylic strip throughout the observation chamber. The strip was used to restrict the movement of the bee against the bottom border of the chamber without causing any harm, to place the Varroa mite on her body through the gate.

Adult Varroa mites were collected from highly infested colonies. A frame covered with bees of these colonies was introduced in a wooden nuc box with the top and bottom replaced by a metallic mesh with 3.0 mm square openings. The nuc box was placed inside a hermetic plastic box to anesthetize the bees and mites by exposing them to carbon dioxide (CO_2_) for five minutes; a white cardboard sheet was placed at the bottom of the box to recover the mites that fell during the procedure. Once the bees were anesthetized, the nuc box was slightly shaken over the cardboard sheet to recover the mites that remained inside the nuc box. The mites were recovered from the cardboard sheet using a fine brush and transferred to a Petri dish containing drone pupae, and the Petri dishes were placed in an incubator at a temperature of 36 °C and relative humidity of 60% to ensure the survival of the mites.

To evaluate the individual honeybee grooming behavior of the colonies, 35 to 38 workers from each colony were collected from the central frame of the brood chamber and transferred to Benton cages with candy. The cages were placed in an incubator at a temperature of 36 °C and relative humidity of 60%.

The behavioral assay to measure the individual honeybee grooming behavior of each worker was conducted by two observers. A worker was introduced into the observation chamber through the gate on the acrylic panel and was immobilized using the acrylic strip, a mite was placed on the thorax of the worker using a fine brush, the gate was closed, and the worker was released inside the observation comb; once the bee was released, the time taken by the worker to express grooming behavior was recorded using a chronometer.

To identify if there were differences between the genetic groups for the expression of individual honeybee grooming behavior, an analysis of variance was conducted using a general linear model under a nested design that included the effect of the genetic group, the effect of the colony nested in the genetic group, and the effect of the random error. A Fisher protected LSD test was used to compare the means of the genetic groups.

To identify the genetic effects associated with the expression of individual honeybee grooming behavior, data was analyzed using a linear regression model to find if the distribution of the means of the genetic groups fit an additive effects model, a dominance effects model, or a model that includes both additive and dominance effects. The response variable in the regression was the mean of each genetic group and the explaining variables were the additive and dominance coefficients for each genetic group based on a genetic model that included additive and dominance effects (Table 1) [46,47].

The Akaike information criterion corrected for small sample size (AICc) and the adjusted coefficient of determination (*r*^2^) were used to determine which model better fit the distribution of the means of the genetic groups.

All statistical analyses were performed with JMP Ver. 4.0.4 software.

### 2.3. Effect of Individual Grooming Behavior Colony Genotype on the Population Growth of Varroa

To determine the effect of the individual grooming behavior colony genotype on the population growth of Varroa, the level of infestation that the mite reached in the colonies over a period of six months was analyzed.

Colonies were treated against Varroa mite 30 days after they were established, with plastic strips impregnated with flumethrine (Bayvarol^®^, Bayer, Leverkusen, Germany) for a period of 56 days to ensure that the experimental colonies were at the lowest possible level of mite infestation at the beginning of the study.

Once the treatment was concluded, colonies were allowed to be naturally infested by Varroa mites from this point until the end of the study. No further treatment against the mite was applied to the colonies during the time of the study.

The Varroa mite infestation level of the colonies was determined by the percentage of mite infestation in adult honeybees and was measured at the beginning and at the end of the study period.

At the beginning of the study, 90 days after the colonies were established to allow time for workers in the colony to be replaced by daughters of the single-drone-inseminated queens, the mite infestation level of the colonies was measured by the average level of infestation of three samples of approximately 100 bees collected from the central frames of the brood chamber of each colony, four days after the treatment against the mite was concluded, to confirm that the experimental colonies were at the lowest possible level of mite infestation at the beginning of study.

At the end of the study, the Varroa infestation level was measured by the average level of infestation of three samples of approximately 100 bees collected from the central frames of the brood chamber of each colony 184 days after the treatment against the mite was concluded, to measure the infestation level that Varroa reached in each colony at the end of the period of study.

Honeybee samples were collected in 250 mL plastic jars with 70% ethanol. To determine the level of infestation in each sample, the jars were agitated for 15 min with a laboratory mechanical shaker to separate the mites from the honeybees and the content of each jar was poured into a white container, where mites and workers were separated and counted. The mite infestation was calculated by the proportion of mites in relation to the number of honeybees in the sample.

To identify if there were differences between the genetic groups for the colony’s Varroa infestation level at the end of the study, an analysis of variance was conducted using a general linear model under a complete random design that included the effect of the genetic group and the effect of the random error. A Fisher protected LSD test was used to compare the means of the genetic groups.

The honeybee population size of the colonies was measured at the end of the study, by recording the number of frames with brood and the number of frames covered with bees in each colony. To determine if there were differences in the honeybee colony population size between the genetic groups, a Kruskal–Wallis test was conducted to compare the number of frames covered with bees and the number of frames with brood.

All statistical analyses were conducted with JMP Ver.4.0.4 software.

### 2.4. Relationship Between Colony Individual Grooming Behavior and the Infestation Level of Varroa

To determine if there is a relationship between colony individual grooming behavior and the infestation levels of Varroa, a Spearman correlation analysis was conducted between the mean of each colony for the time it takes a bee to respond in performing grooming behavior after a mite was placed on her body and the level of infestation that the mite reached in the colonies over a period of six months measured as the percentage of mite infestation in adult honeybees. Statistical analyses were conducted with JMP Ver.4.0.4 software.

## 3. Results

### 3.1. Genetic Effects for Individual Honeybee Grooming Behavior

A total of 3974 workers from the 112 honeybee colonies were evaluated with the behavioral assay. Differences in individual honeybee grooming behavior were found between the genetic groups (*F* = 20.83; *df* = 5, 3862; *p* < 0.001) and between the colonies nested in the genetic groups (*F* = 3.01; *df* = 106, 3862; *p* < 0.001).

The bees of the high-grooming-behavior group (HH) responded in performing grooming behavior after a mite was placed on their bodies faster than the bees of the other five genetic groups (*p* < 0.05). There were no differences in the time to respond between the bees of the high-grooming-behavior backcross (BCH) and the bees of the F1 (HL) group (*p* > 0.05), but the bees of the high-grooming-behavior backcross group (BCH) responded faster than the bees of the other three genetic groups included in the study (LH, BCL, and LL) (*p* < 0.05). There were no differences between the bees of the F1 (HL) group, the F1 (LH) group, and the low-grooming-behavior backcross group (BCL) (*p* < 0.05), but the bees of these three genetic groups (HL, LH, and BCL) responded faster than the bees of the low-grooming-behavior group when a mite was placed on their bodies (LL) (*p* < 0.05).

The mean time it took the bees to respond after a Varroa mite was placed on their bodies (± SE) was 26.56 ± 1.04 s for the high-grooming-behavior group (HH), 32.86 ± 1.07 s for the high-grooming-behavior backcross group (BCH), 35.60 ± 1.01 s for F1 (HL) group, 36.23 ± 1.07 for the F1 (LH) group, 37.09 ± 1.04 s for the low-grooming-behavior backcross group (BCL), and 40.62 ± 1.03 s for the low-grooming-behavior group (LL).

The adjusted coefficient of determination and the Akaike information criterion values estimated in the linear regression analysis indicate that the distribution of means of the genetic groups for the expression of individual honeybee grooming behavior fits a genetic additive effects model (*r^2^* = 0.92; *F* = 47.61; *df* = 1, 4; *p* = 0.002) (AICc = 22.32), better than a dominance effects model for low grooming behavior (*r^2^* = 0.83; *F* = 24.98; *df* = 1, 4; *p* = 0.008) (AICc = 25.78), or a dominance effects model for high grooming behavior (*r^2^* = 0.35; *F* = 3.74; *df* = 1, 4; *p* = 0.125) (AICc = 33.71).

**Figure 2:**
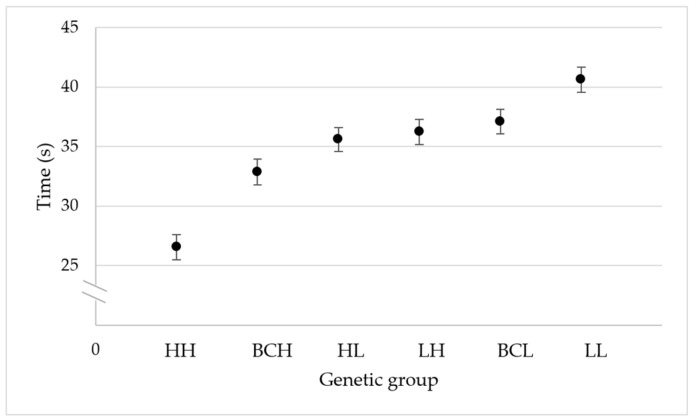
Distribution of average time (±SE) for the expression of individual honeybee grooming behavior of the high-grooming-behavior group (HH), high-grooming-behavior backcross group (BCH), F1 (HL) group, F1 (LH) group, low-grooming-behavior backcross group (BCL), and low-grooming-behavior group (LL). Different letters indicate differences in the average time for the expression of individual honeybee grooming behavior based on an analysis of variance and a Fisher LSD test (*p* < 0.05).

The adjusted coefficient of determination and the Akaike information criterion values estimated in the linear regression analysis indicate that the distribution of means of the genetic groups for the expression of individual honeybee grooming behavior fits a model that includes additive and dominance for high-grooming-behavior effects (*r^2^* = 0.95; *F* = 48.76; *df* = 2, 3; *p* = 0.005) (AICc = 21.60), and a model that includes additive effects and dominance for low-grooming-behavior effects (*r^2^* = 0.95; *F* = 48.76; *df* = 2, 3; *p* = 0.005) (AICc = 21.60) better than a model that only includes additive effects (*r^2^* = 0.92; *F* = 47.61; *df* = 1, 4; *p* = 0.002) (AICc = 22.32) (Figure 2).

In the model that includes additive and dominance for high-grooming-behavior effects, the additive effects were significant (*F* = 48.96; *df* = 1, 3; *p* = 0.006), and the dominance effects for high grooming behavior were not significant (*F* = 4.79; *df* = 1, 3; *p* = 0.116). In the model that includes additive and dominance for low-grooming-behavior effects, the additive effects were significant (*F* = 10.87; *df* = 1, 3; *p* = 0.046), and the dominance effects for low grooming behavior were not significant (*F* = 4.79; *df* = 1, 3; *p* = 0.116).

### 3.2. Effect of Individual Grooming Behavior Colony Genotype on the Population Growth of Varroa

At the beginning of the study, no Varroa infestation was detected in the honeybee samples collected from the colonies after being treated against the mite. The colonies were at the lowest possible level of mite infestation at the beginning of the study.

At the end of the study, differences in the Varroa infestation were detected among the honeybee colonies. There were differences in the population growth of Varroa over a period of six months between the genetic groups (*F* = 31.27; *df* = 5, 107; *p* < 0.001).

The infestation level that Varroa reached after six months in the colonies of the high-grooming-behavior group (HH) was lower than in the other five genetic groups (BCH, HL, LH, BCL, and LL) (*p* < 0.05). The infestation level in the high-grooming-behavior backcross (BCH) colonies was lower than in the colonies of the two F1 (HL and LH), the low-grooming-behavior backcross (BCL), and the low-grooming-behavior (LL) groups (*p* < 0.05). There were no differences in the Varroa infestation level between the two reciprocal F1 groups (HL) and (LH) (*p* > 0.05), but these two groups had lower infestation levels than the low-grooming-behavior backcross (BCL) and the low-grooming-behavior groups (LL) (*p* < 0.05). The Varroa infestation level in the low-grooming-behavior backcross group (BCL) was lower than in the low-grooming-behavior group (LL) (*p* < 0.05).

**Figure 3:**
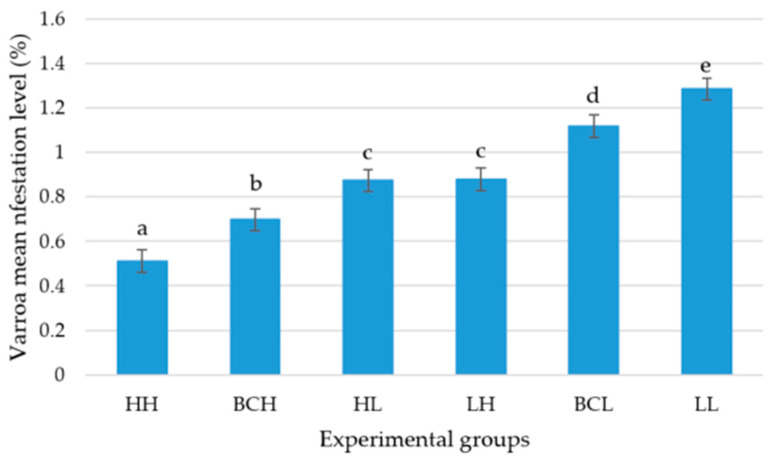
Varroa mean infestation level (± SE) of the high-grooming-behavior group (HH), high-grooming-behavior backcross group (BCH), F1 (HL) group, F1 (LH) group, low-grooming-behavior backcross group (BCL), and low-grooming-behavior group (LL). Different letters indicate differences in the mean level of Varroa infestation based on an analysis of variance and a Fisher LSD test (*p* < 0.05).

The mean level of infestation (± SE) that the mite reached over a period of six months in the colonies of the high-grooming-behavior group (HH) was 0.512% ± 0.051, in the high-grooming-behavior backcross group (BCH) 0.699% ± 0.049, in the colonies of the F1 (HL) group 0.874% ± 0.048, in the F1 (LH) group 0.881% ± 0.051, in the low-grooming-behavior backcross group (BCL) 1.118% ± 0.049, and in the colonies of the low-grooming-behavior group (LL) 1.285% ± 0.049 (Figure 3).

### 3.3. Relationship Between Colony Individual Grooming Behavior and Infestation Level of Varroa

**Figure 4:**
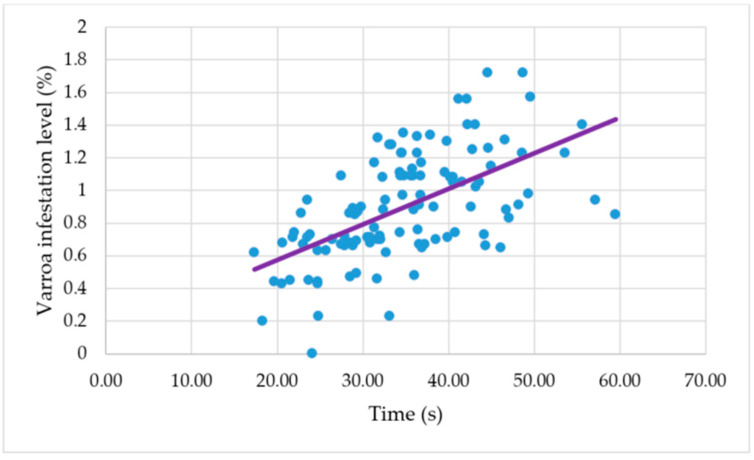
Correlation between the Varroa mean infestation level and the time it takes a bee to respond in performing grooming behavior after a mite was placed on her body.

A positive correlation was found between the mean colony time it takes a bee to respond in performing grooming behavior after a mite was placed on her body and the level of infestation that Varroa reached in the colonies after six months, measured as the percentage of mite infestation in adult honeybees (*r* = 0.57; *n* = 112; *p* < 0.001) (Figure 4).

## 4. Discussion

The results of the study indicate that there were differences between the genetic groups in the expression of honeybee individual grooming behavior and that both additive genetic effects and dominance genetic effects are involved in the expression of this behavioral trait.

The results of the study also indicate that there were differences between the genetic groups in the level of infestation of Varroa in the colonies and that there was a correlation between the individual honeybee grooming behavior of the colonies and the level of infestation of the mite in the colonies.

The results indicate that additive genetic effects explain a major proportion of the distribution of the means of the genetic groups for the expression of individual honeybee grooming, followed by dominance genetic effects for low grooming behavior and by dominance genetic effects for high grooming behavior.

The presence of additive genetic effects in the expression of individual honeybee grooming behavior indicates that quantitative genetic breeding methods can be used to develop high individual honeybee grooming behavior genotypes. The breeding methods will need to consider the presence of the dominance effects detected for the expression of this behavior.

The differences found between the genetic groups suggest that a selection process to breed for high individual honeybee grooming will lead to the development of honeybee genotypes that will be significantly different from unselected honeybee genotypes for the expression of the trait.

Based on the results of our study, and since there is a QTL (*groom-1*) for the expression of individual honeybee grooming behavior on honeybee chromosome five [39], and that genes that are located within the genomic region of the QTL, and in other regions of the honeybee genome, have been associated with the expression of the trait [39,40,41], breeding programs that include both quantitative genetics methods and marked-assisted methods could be designed to develop high-individual-honeybee-grooming-behavior honeybee lines, if molecular markers linked to the QTL and to the genes are developed to identify honeybee genotypes for high grooming behavior.

The honeybee colony population used in this study was an experimental population, composed of six segregating genetic groups. The population was developed to study the genetic effects associated with the expression of individual honeybee grooming behavior. The queens that lead the colonies were single-drone-inseminated, which means that the colonies were composed of only one family of bees.

Since the genetic effects associated with the expression of individual honeybee grooming behavior found in this study were additive and dominance, it can be assumed that, in a colony composed of many families, as it would be when a queen naturally mates with many drones or when a queen is instrumentally inseminated with the semen of more than one drone, the expression of the individual grooming behavior of the colony will be the sum of the genotypes of each family for the part of the trait that is regulated by additive effects, and by the interactions of the genotypes of the families for the part of the trait that is regulated by dominance effects. This needs to be tested in further studies conducted to understand how the interactions between honeybee families with different genotypes for individual grooming behavior affect the colony expression of this trait.

The differences found between the genetic groups for the levels of infestation that Varroa reached in the colonies indicate that there is a relationship between the individual grooming behavior genotype of the colonies and the population growth of Varroa. The high-grooming-behavior (HH) and the backcross high-grooming-behavior (BCH) genetic groups had lower infestation levels than the two hybrid groups (HL and LH) and these groups had lower infestation levels than the backcross low-grooming (BCL) and low-grooming-behavior (LL) groups. These results suggest that there is an effect of the individual grooming behavior genotype of the colonies on the resistance of honeybee colonies to the population growth of Varroa.

Since the experimental colonies were headed by single-drone-inseminated queens, the level of infestation that Varroa reached in the colonies was measured over a period of six months; even though this could be considered a relatively short period of time, we were able to detect differences in the colony population growth of Varroa, due to the effect of the genetic group of the colonies. It is possible that these differences would be more evident if we were able to measure the population growth of the mite for a longer period of time. Previous studies have shown that differences between colonies could be detected in the infestation levels of Varroa over relatively short periods of time [39], particularly if control is taken to ensure that the colonies were free of the mite at the beginning of the studies, and that the colonies are exposed to the same probability to be infested by the mite, as was carried out in this study.

The positive correlation found between the individual honeybee grooming behavior of the colonies and the Varroa levels of infestation in the colonies indicates that there is a relation between the expression of individual honeybee grooming behavior and Varroa infestation levels. The colonies where the honeybees responded faster in performing grooming behavior after a mite was placed on their bodies had lower levels of mite infestation.

This is the first study where a relationship has been found between the honeybee individual grooming behavior of the colonies and the infestation level of Varroa in the colonies. There is only one previous study that searched for this relationship [31], but no correlation was found between these two variables in that study. The main differences that could lead to the different results between the two studies are in the way individual grooming behavior was measured, in the structure of the honeybee populations included, in the way that the infestation level of Varroa was measured, and in the experimental control of the mite infestation in the colonies.

In the previous study [31], individual grooming behavior was measured by rating the intensity of the grooming response of worker bees that are artificially infested with a mite [31,40], and by recording if artificially infested bees dislodge the mites from their bodies by grooming [31,40]. In our study, individual grooming behavior was measured by recording the time it takes for a bee to respond in performing grooming behavior after a mite is placed on her body [39].

In terms of the structure of the honeybee populations, in the previous study [31], four independent experiments were reported. In each experiment, two genetic groups were compared, none of the genetic groups included were selected for the expression of individual honeybee grooming behavior and a relatively small number of colonies were included in each experiment (8 to 18 colonies). In this study, the honeybee population was composed of six segregating genetic groups that were derived from two honeybee lines, one selected for high individual honeybee grooming behavior and one selected for low individual honeybee grooming behavior, and a relatively large number of colonies were included in the study (*n* = 112).

In the previous study [31], the mite infestation level in the colonies was measured by the number of mites fallen per day on the bottom boards of the colonies and no experimental control of the mite infestation was conducted; it is reported that the colonies did not receive any treatment against Varroa for at least one year before the beginning of the study. In our study, the infestation level of Varroa in the colonies was measured by the percentage of mite infestation in adult honeybees and experimental procedures were conducted to study the levels of infestation of Varroa. The colonies were free of the mite at the beginning of the study and the infestation level that Varroa reached in the colonies over a period of 184 days was the variable measured in the study.

The additive and dominance genetic effects for the expression of individual honeybee grooming behavior; the effect of the individual grooming behavior genotype of the colonies on the resistance of the colonies to the population growth of Varroa; and the relationship of the levels of Varroa infestation in the colonies with the colony expression of individual grooming behavior, that were found in this study, are related to the way that grooming behavior was measured. In this study, individual honeybee grooming behavior was measured by recording the time it takes for a bee to respond in performing grooming behavior after a mite is placed on her body [39]. Individual honeybee grooming behavior is also measured by rating the intensity of the grooming response of worker bees that are artificially infested with a mite [31,40], and by recording if artificially infested bees dislodge the mites from their bodies by grooming [31,40]. Studies need to be conducted to determine if our findings are valid when other methods are used to measure the trait.

Honeybee mite grooming behavior is divided into autogrooming and allogrooming [28,33,35,36]; individual honeybee grooming behavior basically refers to autogrooming, that is, when a worker bee grooms herself with the legs to get rid of a mite, so the findings of our study basically apply to honeybee autogrooming. It is important to consider that the time it takes for a bee to respond in performing grooming behavior after a mite is placed on her body is one of the components of honeybee autogrooming, and the behavior is not complete until the bee removes the mite from her body.

The results of this study suggest that, since additive and dominance genetic effects are associated with honeybee individual grooming behavior and since this behavioral trait has an effect on the levels of Varroa infestation in the colonies, breeding programs can be conducted to develop high-individual-honeybee-grooming-behavior honeybee lines that will show a degree of resistance to the population growth of Varroa in the colonies.

## Figures and Tables

**Table 1:** Coefficients used in the analysis of regression to identify the genetic effects associated with the expression of individual honeybee grooming behavior.

Genetic Group	Coefficients		
	Additive Effects	High Grooming Dominance Effects	Low Grooming Dominance Effects
HH	−1	−1	−1
BCH	−0.5	−1	0
HL	0	−1	1
LH	0	−1	1
BCL	0.5	0	1
LL	1	1	1

## Data Availability

The data analyzed in the study can be provided by the corresponding author upon reasonable request.
